# Non-Invasive Assessment of Endothelial Dysfunction in Pulmonary Arterial Hypertension

**DOI:** 10.31138/mjr.32.1.6

**Published:** 2021-02-15

**Authors:** Athanasia Dara, Alexandra Arvanitaki, Marieta Theodorakopoulou, Christos Athanasiou, Eleni Pagkopoulou, Afroditi Boutou

**Affiliations:** 1 Fourth Department of Internal Medicine, Hippokration University Hospital, Medical School, Aristotle University of Thessaloniki, Thessaloniki, Greece; 2 First Department of Cardiology, AHEPA University Hospital, Medical School, Aristotle University of Thessaloniki, Thessaloniki, Greece; 3 Adult Congenital Heart Centre and National Centre for Pulmonary Arterial Hypertension, Royal Brompton Hospital, Guy’s and St Thomas’ NHS Foundation Trust, Imperial College, London, UK; 4 Department of Internal Medicine, General Hospital of Giannitsa, Giannitsa, Greece; 5 Department of Respiratory Medicine, G. Papanikolaou Hospital, Thessaloniki, Greece

**Keywords:** pulmonary arterial hypertension, microvasculature, endothelial dysfunction, flow mediated dilation, arterial stiffness, nailfold videocapillaroscopy, near-infrared spectroscopy, magnetic resonance perfusion imaging, biochemical markers

## Abstract

Pulmonary arterial hypertension (PAH) is characterised by an increased pressure in the pulmonary arterial circulation, resulting in the elevation of pulmonary vascular resistance. Pulmonary endothelial dysfunction and inflammation, triggered by shear stress and hypoxia, constitute the hallmarks of pulmonary vasculopathy by promoting endothelial and smooth muscle cells proliferation, vasoconstriction, and thrombosis. While research was predominantly focused on pulmonary vasculature, the investigation of peripheral endothelial damage in different vascular beds has attracted the interest over the last years. As a result, effective non-invasive methods that can assess the endothelial function and the architectural integrity have been utilized for the evaluation of pulmonary and peripheral vasculature. Non-invasive plethysmography, pulmonary flow reserve, nailfold videocapillaroscopy, near-infrared spectroscopy, and imaging techniques such as magnetic resonance angiography and perfusion imaging coupled by a number of biomarkers can be used for the assessment of peripheral vascular function in PAH individuals. In this review, we summarise and critically approach the current evidence of more systemic derangement of vascular function in PAH defined by novel, non-invasive methods employed for functional and morphological assessment of endothelium and microcirculation.

## INTRODUCTION

Pulmonary arterial hypertension (PAH) is haemodynamically defined by a mean pulmonary arterial pressure (mPAP)>20 mmHg at rest, a pulmonary capillary wedge pressure (PCWP)≤15 mmHg, and an increased pulmonary vascular resistance (>3 Woods Unit, WU) according to the 6^th^ World Symposium of Pulmonary Hypertension.^1^ PAH is a serious complication in connective tissue disorders (CTD-PAH) especially in systemic sclerosis (SSc), mixed connective tissue disease (MCTD), dermatomyositis and systemic lupus erythematosus (SLE). In fact, CTD-PAH is the second most common cause of PAH after idiopathic PAH, representing one of the leading causes of death in this population.^2^ Beyond common pathogenetic mechanisms with other forms of PAH such as thrombosis, vasoconstriction, endothelial, and smooth muscle cells proliferation, a more pronounced inflammatory and autoimmune component has been described in CTD-PAH.^3^ Infiltrating macrophages and lymphocytes, antinuclear antibodies, rheumatoid factor, and proinflammatory cytokines have been detected in the pulmonary vessels, as well as the serum of patients with CTD-PAH.^4,5^ The clinical evidence of such observations suggests that early administration of corticosteroids and immunosuppressants in SLE and MCTD patients could prevent irreversible pathologic changes in pulmonary vessels and improve functional status.^6^

### 

#### The endothelium and its role in regulation of vascular tone

The endothelium, the inner cellular lining of blood and lymphatic vessels, plays a key role in the regulation of vascular tone, permeability, coagulation, inflammation, and vascular homeostasis in response to various stimuli.^7^ The arterial wall is comprised of three cellular layers: the tunica adventitia, the tunica media, and the tunica intima. The region enclosed by the vessel wall is called the vessel lumen. Exposure to genetic, as well as environmental factors, such as hypoxia, induces vascular remodelling in PAH; namely, smooth muscle cell proliferation in the tunica media and enhancement of endothelial cell apoptosis in the tunica intima. Endothelial-like cells, when proliferated in a disorganized manner, form glomeruloid-like lesions called “plexiform lesions”.^8^ Tuder et al. demonstrated that these lesions are responsible for the expression of vascular endothelial growth factor, and the hypoxia inducible factor -1a.^8^ Data collected from 62 PAH patients indicated that plexiform lesions can be detected in the lungs of up to 90% of PAH patients.^9^ Three-dimensional reconstructive studies were able to show another onion-skin arrangement of endothelial- and/or smooth muscle cells, situated in proximity to plexiform lesions.^10^ This cellular accumulation was characterised as an “obliterative concentric lesion”, and such lesions appear to be completely restricted to the pulmonary vascular lumen.

Impairment of endothelial function in pulmonary circulation is the initial and fundamental event in the development and progression of PAH. In particular, dysregulated synthesis and release of vasodilator mediators such as nitric oxide (NO) and prostacyclin accompanied by overexpression of vasoconstrictors, such as endothelin-1 culminate in vascular changes typically observed in pulmonary vasculature.^11^ Over the last years the interest has shifted towards the investigation of more systemic peripheral involvement of macro- and micro-circulation in patients with PAH. On the top of validated methods assessing structural and functional parameters of vascular health including flow mediated dilation (FMD) and pulse wave analysis, a number of novel sophisticated modalities such as nailfold videocapillaroscopy (NVC) and near-infrared spectroscopy (NIRS) have emerged for the evaluation of peripheral microcirculation with promising implications in PAH.^12^

The current review provides a thorough report of the available data regarding systemic vascular injury, macrovascular dysfunction and microcirculation changes in peripheral vascular branches in patients with PAH, assessed by non-invasive techniques. The potential utility of biomarkers of endothelial dysfunction as a part of non-invasive evaluation of generalized vasculopathy in subjects with PAH is also briefly discussed.

### Search strategy

A MEDLINE and EMBASE search was carried out according to published guidance on narrative reviews, using the following terms: pulmonary arterial hypertension, pulmonary hypertension, idiopathic pulmonary hypertension, connective tissue related-PAH, systemic sclerosis-related PAH, endothelial dysfunction, pulse wave velocity, flow mediated dilation, nailfold videocapillaroscopy, and near-infrared spectroscopy.^13^ Original research papers and review articles registered until the end of December 2020 were selected to be included in this review. Publications not in English and data from ongoing research were excluded.

## MACROVASCULAR ENDOTHELIAL DYSFUNCTION

The presence of a more generalised peripheral impairment of endothelial homeostasis in PAH was originally determined by measurements of peripheral endothelium-dependent vasoreactivity using FMD, after brachial artery occlusion in individuals with idiopathic and SSc-related PAH, as well as patients with chronic thromboembolic pulmonary hypertension (CTEPH). Peled et al. reported abnormal endothelial function in all PAH groups compared to controls.^14^ Interestingly, the hyperaemic response was significantly correlated with indices of disease severity; namely New York Heart Association (NYHA) classification, mean pulmonary pressure, six-minute walking distance, and arterial oxygen desaturation upon exercise. The generalized vasculopathy in patients with idiopathic and SSc-related PAH was confirmed in a later study with a larger number of participants, which again demonstrated endothelial dysfunction in PAH individuals compared to controls, while a trend towards increased arterial stiffness in SSc subgroup was also noticed.^15^ Finally, Wolff et al. found that endothelial dysfunction in peripheral, non-pulmonary conduit vessels assessed by FMD in idiopathic PAH was correlated with acute pulmonary vasoreactivity defined as a positive pulmonary vascular response to inhaled iloprost during right heart catheterization (RHC).^16^ The later provides a link between systemic vascular injury and hemodynamic parameters supporting the hypothesis that at least to some extent, PAH can be perceived as a systemic vascular disorder affecting both the pulmonary and the peripheral vasculature.

## MICROVASCULAR ENDOTHELIAL DYSFUNCTION

### Pulmonary microcirculation

Early pulmonary vascular disease is often clinically silent, as initial pulmonary microvascular damage does not lead to significant changes in the pulmonary arterial pressure. Subsequently, there is an increasing interest in assessing the status of pulmonary microcirculation in early stages before advanced alterations occur. Zimmermann et al. attempted to determine the degree of pulmonary endothelial dysfunction in children with IPAH by using the pulmonary flow reserve method.^17^ The pulmonary flow reserve was determined by acetylcholine infusion into segmental pulmonary arteries using quantitative angiography and an intra-arterial Doppler flow wire in 17 children with IPAH. Endothelial function was then assessed by classifying the reactivity of the pulmonary to systemic arterial pressure ratio, short-term oxygen and intravenous epoprostenol or aerosolized iloprost administration. The results indicated that acetylcholine infusion can cast a light on the pathogenesis of IPAH providing valuable information regarding the prognosis of the disease in patients with impaired vasoreactivity.

Another method for evaluating the pulmonary micro-circulation is based on the assessment of the peak hyperaemic flow using the thermodilution derived mean transit-time. Results from a study performed on baboons, in which peak hyperaemic flow was assessed using sensor-guidewires and adenosine/papaverine as hyperaemic agents, showed that these novel indices can detect progressive pulmonary microvascular degeneration and can contribute to the assessment of early pulmonary vascular disease.^18^ Therefore, it becomes apparent that pulmonary flow shows promising results for the evaluation of endothelial activity by measuring vasoreactivity. However, this method is still in its infancy and further studies in humans are needed to validate these early findings.

### Systemic microcirculation

#### Nailfold videocapillaroscopy

Nailfold videocapillaroscopy is a simple, non-invasive method used to recognize morphological and/or functional abnormalities in the peripheral microvasculature. It was initially developed for the diagnostic assessment of patients with Raynaud’s syndrome and is currently included in the European League Against Rheumatism (EULAR) and the American College of Rheumatology (ACR) update of the classification criteria for SSc.^19^ The most common parameters assessed are capillary density per linear millimetre, capillary dimensions (mainly capillary loop width), the presence of microhaemorrhages and capillary shape abnormalities.^20^ Given its ability to visualise different stages of peripheral vasculopathy, NVC is considered as a surrogate marker of SSc severity and progression, including vascular impediments such as PAH and/or visceral organ involvement.^21^ Similarly, in PAH, peripheral microangiopathy –assessed by NVC– may reflect the progression of pulmonary vasculopathy and provide a non-invasive, indirect tool for the evaluation of the disease itself.^22^ For example, a study including 12 SSc-related PAH patients and 12 SSc patients without PAH found a significant correlation between mPAP and worse NVC score, defined by the presence of enlarged, giant, ramified, or bushy capillaries, microhaemorrhages, and loss of capillaries.^23^ Abnormal capillaroscopic pattern has also been correlated with pulmonary vascular resistance in SSc-related PAH.^24^

Another study in 66 consecutive patients aimed to relate NVC scleroderma patterns classified into “normal”, “early”, “active” or “late” with organ involvement.^25^ This terminology, used to classify NVC abnormalities amongst SSc subjects, depicts distinct patterns visualised at different stages of the disease. For instance, “early” changes are characterised by the presence of few giant capillaries and microhaemorrhages, whereas “late” changes include progressive capillary loss and disorganized capillaries. This study indicated a strong correlation between NVC patterns and future severe vascular and lung involvement according to worsening scleroderma patterns. A recent meta-analysis confirmed the notable associations between SSc-PAH, capillary density and NVC pattern suggesting the potential integration of NVC into the current workout for the early detection of vascular injury in patients with CTDs.^26^

**Figure 1.**
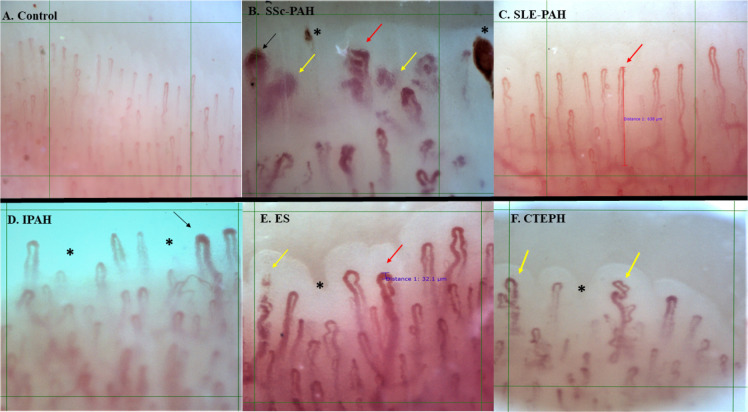
Nailfold video-capillaroscopic features in patients with various types of precapillary pulmonary hypertension. **A.** Healthy control; normal capillary density (10 loops/mm), hairpin-shaped capillaries with normal loop width. **B.** PAH associated with systemic sclerosis (SSc-PAH); active scleroderma pattern with reduced capillary density (5 capillary loops/mm), giant capillaries (black arrow), disorganized capillaries (yellow arrows), abnormal capillary shapes (red arrow) and microhemmorhages (asterisks). **C.** PAH associated with systemic lupus erythematosus (SLE-PAH); reduced capillary density (6 loops/mm) with elongated capillaries (>500μm, red arrow) **D.** Idiopathic pulmonary arterial hypertension (IPAH); reduced capillary density (5 capillary loops/mm), avascular areas (asterisk) and irregularly enlarged capillaries (> 20μm and <50μm, arrow). **E.** Eisenmenger syndrome (ES); reduced capillary density (6 loops/mm), avascular area (asterisk), enlarged loop diameter (32.1μm, red arrow) and microhemorrhage (yellow arrow). **F.** Chronic thromboembolic pulmonary hypertension (CTEPH); mildly reduced capillary density (7 capillary loops/mm), avascular areas (asterisk) and abnormal capillary shapes (yellow arrows). Grid pattern represents area of 1mm^2^. Magnification 200×. All images were retrieved from the record of the Fourth Department of Internal Medicine, Hippokration General Hospital, Aristotle University of Thessaloniki. Informed consent was obtained from patients to publish their images.

In the context of PAH of other aetiologies, a number of studies have investigated NVC alterations in patients with idiopathic PAH. Corrado et al. studied 39 SSc patients (19 with PAH), 21 subjects with idiopathic PAH, and 20 healthy subjects.^27^ Apart from the changes detected in SSc-PAH, they also demonstrated increased capillary width in patients with idiopathic PAH patients compared to healthy controls. The bidirectional relationship between peripheral microvascular remodelling and PAH was also examined by Hofstee et al., who described a decreased capillary density in both SSc-PAH and IPAH individuals, presenting such changes as an indicator of disease severity.^28^ Last but not least, recent reports including patients with idiopathic PAH and Eisenmenger syndrome have revealed abnormal capillaroscopic patterns across a wider spectrum of groups with pulmonary vascular disease.^29^ Finally, a recent study demonstrated significant NVC changes, such as reduction in capillary density and increased capillary dimensions in patients with IPAH and CTEPH. More than half of patients with IPAH presented microhaemorrhages on capillary nailfold, while shape abnormalities and capillary thrombi were detected at a greater extent in patients with CTEPH.^30^ This study is part of a larger prospective study focusing on the documentation of peripheral microangiopathy in patients with pre-capillary PH (including patients with IPAH, CTD-PAH, Eisenmenger syndrome and CTEPH), as well as the association of qualitative and quantitative NVC parameters with clinical, haemodynamic, echocardiographic, biochemical and functional parameters of disease severity in PH patients (**Figure 1**).^31^

#### Near-infrared spectroscopy

The increased vascular resistance in PAH leads to a disturbance in oxygen delivery to the periphery and an impairment in tissue oxygenation. The balance between oxygen supply and demand is reflected in the index mixed venous oxygen saturation (SvO_2_), which is typically reduced in PAH patients.^32^ Spatially resolved NIRS provides a non-invasive and rapidly responsive method for assessing skeletal muscle oxygenation. This is accomplished by measuring absorption differences in the near-infrared spectrum of light between oxy- and deoxy-haemoglobin and myoglobin molecules in the microvasculature. The tissue oxygenation index (StO_2_) is adopted for the depiction of the dynamic balance between local tissue oxygen supply and utilization in all subjects.^33,34^ Since the contribution of myoglobin to the NIRS signal is not critical, StO_2_ is generally used as the ratio of oxygenated to total tissue haemoglobin concentration. A study by Panagiotou et al. correlated measurements of StO_2_ with SvO_2_ and venous oxygen saturation in the inferior vena cava during RHC to evaluate the use of NIRS in PAH.^35^ For this purpose, 25 PAH patients were assessed at rest, whereas 10 of those subjects were also examined during exercise. Results showed that skeletal muscle StO_2_ in PAH subjects correlated considerably with SvO_2_ under resting and exercise conditions and furthermore, it is possible that this could indicate disease severity. Therefore, NIRS could serve as a non-invasive tool for the qualitative evaluation of the dynamic balance between oxygen supply and demand in PAH as well as for the assessment of disease progression and severity. Inspired by the work of Panagiotou et al., NIRS was used to confirm the significant contribution of peripheral muscle alterations to the exercise intolerance in PAH patients.^36^ Finally, Dimopoulos et al. assessed peripheral muscle microcirculation by utilising NIRS in 8 PAH patients, 8 matched healthy subjects (controls), and 16 patients with chronic heart failure.^37^ Tissue O_2_ saturation was measured on the thenar muscle as the haemoglobin saturation (%) in the microvasculature compartments. Subsequently, the 3-min brachial artery occlusion technique was applied before, during and after 15 min of breathing 100% O_2_. The oxygen consumption rate, the reactive hyperaemia time, and the time needed for tissue O_2_ saturation to reach its baseline value after the release of the occlusion were measured. PAH subjects presented a significant impairment of peripheral muscle microcirculation, decreased tissue O_2_ saturation (possibly attributed to hypoxemia), slower reactive hyperaemia time (evidence of endothelial dysfunction) and peripheral systemic vasoconstriction. Finally, acute hyperoxic breathing improved resting tissue O_2_ saturation and reduced the oxygen consumption rate and reactive hyperaemia time during reperfusion, probably due to increased oxidative stress and evoked vasoconstriction.^38^ These findings demonstrate an imbalance in oxygen supply and demand in peripheral muscles in patients with PAH, a phenomenon which may contribute to exercise intolerance in this population.

#### Imaging Techniques

Shifting the focus from the visualisation of the larger vessels (macrocirculation) down to the segmental branches and the effect of the contrast agent during its passage through the arterioles and the capillary bed (microcirculation) paved the way to the development of perfusion magnetic resonance imaging (MRI).^39,40^ The direct visualisation of ventilation was achieved using dedicated tracer gases, such as hyperpolarised noble gases (helium-3 and xenon-129). For example, Oxygen-sensitive He-3 MRI allows for quantification of the alveolar partial pressure oxygen, which can serve as an important complement for comprehensive ventilation-perfusion MRI. However, it has yet to be approved for clinical use as an MRI contrast agent by the FDA mainly due to limited availability (it is a by-product of tritium decay and is therefore classified as a strategic product in the United States). In the case of PH, MR angiography and MR perfusion imaging allow an in-depth evaluation of the localisation of the chronic thromboembolic material (underlying cause of PH).^41^ Moreover, perfusion MRI can be quantitatively evaluated in order to assess the severity of the small vessel degeneration. Perfusion MRI is a sensitive surrogate for monitoring the disease, while structural imaging of the lung allows the exclusion of other parenchymal diseases. Lastly, measurements of blood flow and right heart structural modifications provide estimates for changes in pulmonary arterial pressure as well as indications of cardiac strain and associated valvular disease.^42^

A study conducted by Ohno et al. aimed at the quantitative assessment of regional pulmonary perfusion in the entire lung, using 3D ultrafast dynamic contrast-enhanced MRI in 40 volunteers (15 healthy, 25 PAH patients).^43^ Quantitative pulmonary perfusion parameters such as pulmonary blood flow, mean transit time and pulmonary blood volume showed significant differences between normal volunteers and PAH patients. Thus, it is possible that three-dimensional ultrafast dynamic contrast-enhanced MR imaging can be used for the assessment of pulmonary perfusion parameters in the entire lung of both normotensive and hypertensive patients. Consequently, MRI techniques can be used for the visualization of the pulmonary vascular bed and for the assessment of pulmonary perfusion parameters but at this moment its sensitivity is limited due to lack of a highly available and effective contrast agent.

## BIOCHEMICAL MARKERS OF ENDOTHELIAL DYSFUNCTION

The detection and quantification of circulatory molecules that are correlated with endothelial degeneration and dysfunction are useful for the early detection and monitoring of PAH. Biomarkers can be roughly categorised according to their involvement in endothelial cell dysfunction, inflammation, epigenetics, cardiac function, oxidative stress, metabolism and extracellular matrix.^44^ A potential endothelial cell marker is asymmetric dimethylarginine (ADMA), an endogenous inhibitor of nitric oxide (NO) synthase. Impairment of the enzymatic activity of endothelial NO synthase leading to reduced bioavailability of NO is considered one of the main pathophysiologic mechanisms of PAH and is mainly mediated by ADMA. ADMA metabolism is dysregulated in PAH and high serum ADMA levels can be a useful biomarker for PAH progression.^45,46^ A study by Giannakoulas et al. also underlined the importance of ADMA in the assessment of pulmonary endothelial dysfunction associating it with changes in the expression and activity of connexin.^47^

**Table 1.** Non-invasive techniques for the assessment of peripheral macro- and microvascular dysfunction in PAH.

**Study**	**Sample**	**Method**	**Objectives**	**Evaluated** **Variables**	**Results**
**Peled et al**.^13^(2008)	**54 patients** -28 IPAH -10 SSc-PAH -7 CTEPH -9 Eisenmenger **21 control subjects**	Plethysmograph EndoPAT 2000 Post-occlusion branchial artery FMD	Systemic endothelial dysfunction and disease severity	-PAT ratio -NYHA classification -6-MWD -O_2_ desaturation on effort -Pulmonary pressure	PAT ratio↓ in all groups except Eisenmenger PAT ratio associated with NYHA, 6MWD, O_2_ des., pulm. pressure
**Peled et al**.^14^ (2009)	**38 patients** -28 IPAH -10 SSc-PAH **21 control subjects**	Fingertip tonometry Plethysmograph EndoPAT 2000 Post-occlusion branchial artery FMD	Arterial stiffness and endothelial dysfunction	-AI -PAT ratio	↓ PAT ratio in IPAH and SSc-PAH Arterial stiffness ↑ in SSc No correlation between AI and PAT ratio
**Wolff et al.**^15^ (2007)	18 IPAH patients 36 control subjects	FMD	Endothelial dysfunction and acute vasoreactivity response	-FMD -mean PAP PVR post inhaled iloprost	↓ FMD in IPAH vs. controls Iloprost ↓ mean PAP and PVR Correlation of FMD with PVR and mean PAP post iloprost administration
**Panagiotou et al.**^34^ (2016)	**25 patients** -13 IPAH -1 HPAH -7 CTD-PAH -1 CHD-PAH -3 PP-PAH	NIRS	Peripheral muscle microcirculation impairment and correlation with disease severity	-StO_2_% -SvO_2_% -SivcO_2_%	StO_2_% correlation with SvO_2_% and with SivcO_2_% SivcO_2_% correlation with SvO_2_%
**Dimopoulos et al.**^36^ (2013)	**32 subjects** -8 PAH patients -8 healthy controls -16 subjects with chronic heart failure	NIRS	Peripheral muscle microcirculation impairment	-StO_2_% -O_2_ consumption rate (%/min) -Reactive hyperemia time	↓ resting StO_2_% and ↑ reactive hyperemia time in PAH subjects
**Hatabu et al.**^37^ (1996)	**8 subjects total (healthy)** - porcine model of pulmonary embolism	MRI	MRI of lung perfusion feasibility	Signal intensity of the lung parenchyma	Parenchymal enhancement Recirculation Pulmonary embolus visible
**Ohno et al**.^41^ (2004)	**40 subjects total** -25 PAH patients -15 healthy controls	MRI	3-D ultrafast dynamic contrast-enhanced MRI feasibility	PBF MTT PBV	Significant alterations

AI: augmentation index; D_O2_: estimated systemic oxygen delivery; CTD-PAH: pulmonary arterial hypertension associated with connective tissue disease; CTEPH: chronic thromboembolic pulmonary hypertension; HPAH: hereditary pulmonary arterial hypertension; IPAH: idiopathic pulmonary arterial hypertension; FMD: flow mediated dilatation; MRI: magnetic resonance imaging; MTT: mean transit time; 6MWD: six-minute walking distance; NIRS: near infrared spectroscopy; NYHA: New York Heart Association; PP-PAH: portopulmonary arterial hypertension; PAT: peripheral arterial tone; PAH: pulmonary arterial hypertension; mean PAP: mean pulmonary arterial pressure; PBF: pulmonary blood flow; PBV: pulmonary blood volume; PVR: pulmonary vascular resistance; StO_2%_: tissue oxygenation index; SvO_2%_: venous oxygen saturation; SivcO_2%_: venous oxygen saturation in the inferior vena cava.

Another inflammatory circulating molecule that could hold value as a biomarker is galectin-3. Galectin-3 functions as an endocrine and paracrine factor for the stimulation of macrophages, fibroblasts, and inflammatory cells. An increase in galectin-3 concentration has been found in patients with left heart failure where it seems to play an important role in cardiac fibrosis and remodeling.^48^ The correlation between galectin-3 and PAH was studied in 15 patients with PAH who underwent echocardiography and a blood test to measure galectin-3 concentrations. The results showed an elevated galectin-3 in all PAH patients.^49^ Although these findings were encouraging, galectin-3 lacks the specificity to serve as a sole biomarker, since elevated concentrations can also be detected in patients diagnosed with renal failure, pulmonary and hepatic fibrosis.^50^

Circulating factors that regulate angiogenesis are also examined for their role as biomarkers in PAH. Malhotra et al. investigated the concentrations of soluble endoglin, vascular endothelial growth factor receptor-1 (sVEGFR1), N-terminal brain natriuretic peptide and C-reactive protein in the peripheral blood of 97 PAH patients.^51^ Moreover, they obtained lung tissue from the aforementioned patients, which they also examined for endoglin expression and microvascular endothelium changes. Expression of these molecules was significantly enhanced in both blood and tissue samples. Tiede et al. confirmed these findings in another study of 76 PAH patients (without however finding a connection between sVEGFR1 and haemodynamic parameters).^52^ sVEGFR1 is likely a modulator of chronic hypoxia-induced pulmonary and peripheral vascular remodelling, which mobilizes endothelial progenitor cells (EPCs). Diller et al. demonstrated that circulating EPCs were markedly reduced in Eisenmenger syndrome and IPAH compared to controls (indicating reduced capacity to maintain endothelial cell homeostasis) and were also negatively associated with inflammatory biomarkers and asymmetric dimethylarginine.^53^ In IPAH, the number of circulating EPCs correlated with invasive hemodynamic parameters and treatment with the phosphodiesterase inhibitor sildenafil was associated with a dose-dependent rise in EPC numbers. In contrast to EPCs, circulating endothelial cells (CECs) are increased in PAH, since they are detached from pulmonary endothelium. Both in PAH associated with congenital heart disease (PAH-CHD) and IPAH, clinical deterioration was associated with increased CEC count, while treatment with PAH-targeted therapy was associated with a decrease.^54^ In addition, CEC count in peripheral blood was 10-fold higher in irreversible versus reversible PAH-CHD and controls.^55^

Another circulating molecule that was found to have a strong correlation with disease severity and mortality in PAH was the potent angiostatic factor, endostatin. Damico et al. discovered a direct association between high serum endostatin concentrations and poor functional status, decreased exercise tolerance, invasive hemodynamic variables and mortality.^56^ Furthermore, research by Kumpers et al. demonstrated that high angiopoietin 2 blood concentration correlates with high pulmonary vascular resistance and is consequently an accurate predictor of survival.^57^ Lastly, Yang et al. detected high ghrelin concentrations in IPAH patients.^58^ Ghrelin is a hormone produced in the gastrointestinal tract responsible for regulating appetite. This hormone also proved to correlate positively with elevations of the pulmonary arterial systolic pressure, plasma N-terminal brain natriuretic peptide, endothelin-1 and nitric oxide levels.

MicroRNAs (miRNAs) as biomarkers and coding genes are responsible for post-transcriptional regulation of gene expression as well as RNA silencing. Their serum concentration can be construed as a direct reflection of existing vascular pathology.^59^ Sarrion et al. studied the role of various miRNAs as potential biomarkers of IPAH.^60^ Out of the miRNAs examined, miR-23a proved to be of special interest due to its association with pulmonary function.

The issue of pulmonary lymphoid neogenesis in IPAH was raised by Perros et al.^61^ Flow cytometry analyses of circulating lymphocytes were used to investigate the systemic mark of pulmonary lymphoid neogenesis. The results of this study demonstrated that IPAH lungs, compared to controls, contained perivascular ectopic (tertiary) lymphoid tissues, as well as B- and T-cell areas with high endothelial venules and dendritic cells. It remains to be seen whether the detection of such auto-antibodies and other lymphoid neogenesis factors, using flow cytometry, could serve as biomarkers for the early detection and diagnosis of IPAH, or if they serve as indicators of a poorer prognosis.

## CONCLUSION

Endothelial dysfunction appears to be the trigger for microvascular degeneration and remodelling in PAH. These vascular changes are no longer considered organ-specific, but have been found to extend to the periphery as well. PAH can present itself as a severe complication of a pre-existing condition, as is the case in many patients with SSc and MCTD. Therefore, finding an available, cost-effective, and non-invasive technique for the evaluation of endothelial function in PAH and other high-risk patients would be of great value for early diagnosis as well as for continuous monitoring of disease progression. Plethysmography and fingertip tonometry for the assessment of endothelial function and arterial stiffness has shown promising results in SSc-PAH and IPAH patients. Furthermore, NVC makes it possible to detect changes in capillary density and dimensions as well as microangiopathy in the periphery directly correlated with disease severity. NIRS provided an explanation for exercise intolerance in PAH patients by demonstrating an imbalance in oxygen supply and demand in skeletal muscles. In more detail, findings in PAH subjects using NIRS include significant impairment of peripheral muscle microcirculation, decreased tissue O_2_ saturation (possibly attributed to hypoxemia), slower reactive hyperaemia time (evidence of endothelial dysfunction) and peripheral systemic vasoconstriction. Additionally, MRI techniques can be used for the visualisation of the pulmonary vasculature and for the assessment of pulmonary perfusion parameters, but the availability of an effective contrast agent is, to date quite limited. Finally, circulating molecules, such as ADMA, galectin-3, ghrelin, MiRNAs and auto-antibodies can be detected and quantified in PAH patients and could serve as biomarkers for disease diagnosis, severity, and progression. While all aforementioned assessment methods are of great promise, further research and clinical studies are required in order to validate their role in an algorithm for a non-invasive detection and monitoring of systemic vascular changes in PAH.

## References

[1] Simonneau G Montani D Celermajer DS Denton CP Gatzoulis MA Krowka M Haemodynamic definitions and updated clinical classification of pulmonary hypertension. Eur Respir J 2019 1 24;53(1):1801913. 30545968 10.1183/13993003.01913-2018 PMC6351336

[2] Arvanitaki A Boutsikou M Anthi A Apostolopoulou S Avgeropoulou A Demerouti E Epidemiology and initial management of pulmonary arterial hypertension: real-world data from the Hellenic pulmOnary hyPertension rEgistry (HOPE). Pulm Circ 2019;9:2045894019877157. 31662847 10.1177/2045894019877157 PMC6792282

[3] Shahane A. Pulmonary hypertension in rheumatic diseases: epidemiology and pathogenesis. Rheumatol Int 2013;33:1655–67. 23334373 10.1007/s00296-012-2659-y

[4] Dorfmuller P Perros F Balabanian K Humbert M. Inflammation in pulmonary arterial hypertension. Eur Respir J 2003;22:358–63. 12952274 10.1183/09031936.03.00038903

[5] Yeo PP Sinniah R. Lupus cor pulmonale with electron microscope and immunofluroescent antibody studies. Ann Rheum Dis 1975;34:457–60. 1241263 10.1136/ard.34.5.457 PMC1006448

[6] Yasuoka H Shirai Y Tamura Y Takeuchi T Kuwana M. Predictors of Favorable Responses to Immunosuppressive Treatment in Pulmonary Arterial Hypertension Associated With Connective Tissue Disease. Circ J 2018;82:546–54. DOI:28904255 10.1253/circj.CJ-17-0351

[7] Tuder RM. Pulmonary vascular remodeling in pulmonary hypertension. Cell Tissue Res 2017;367:643–9. 28025704 10.1007/s00441-016-2539-y PMC5408737

[8] Tuder RM Chacon M Alger L Wang J Taraseviciene-Stewart L Kasahara Y Expression of angiogenesis-related molecules in plexiform lesions in severe pulmonary hypertension: evidence for a process of disordered angiogenesis. J Pathol 2001;195:367–74. 11673836 10.1002/path.953

[9] Stacher E Graham BB Hunt JM Gandjeva A Groshong SD McLaughlin VV Modern age pathology of pulmonary arterial hypertension. Am J Respir Crit Care Med 2012;186:261–72. 22679007 10.1164/rccm.201201-0164OC PMC3886716

[10] Cool CD Stewart JS Werahera P Miller GJ Williams RL Voelkel NF Three-dimensional reconstruction of pulmonary arteries in plexiform pulmonary hypertension using cell-specific markers. Evidence for a dynamic and heterogeneous process of pulmonary endothelial cell growth. Am J Pathol 1999;155:411–9. 10433934 10.1016/S0002-9440(10)65137-1 PMC1866857

[11] Rabinovitch M Guignabert C Humbert M Nicolls MR. Inflammation and immunity in the pathogenesis of pulmonary arterial hypertension. Circ Res 2014;115:165–75. 24951765 10.1161/CIRCRESAHA.113.301141 PMC4097142

[12] Hughes R Tong J Oates C Lordan J Corris PA. Evidence for systemic endothelial dysfunction in patients and first-order relatives with pulmonary arterial hypertension. Chest 2005;128:617S. 16373872 10.1378/chest.128.6_suppl.617S

[13] Gasparyan AY Ayvazyan L Blackmore H Kitas GD. Writing a narrative biomedical review: considerations for authors, peer reviewers, and editors. Rheumatol Int 2011 11;31(11):1409–17. 21800117 10.1007/s00296-011-1999-3

[14] Peled N Bendayan D Shitrit D Fox B Yehoshua L Kramer MR. Peripheral endothelial dysfunction in patients with pulmonary arterial hypertension. Respir Med 2008;102:1791–6. 18678478 10.1016/j.rmed.2008.06.014

[15] Peled N Shitrit D Fox BD Shlomi D Amital A Bendayan D Peripheral arterial stiffness and endothelial dysfunction in idiopathic and scleroderma associated pulmonary arterial hypertension. J Rheumatol 2009;36:970–5. 19369472 10.3899/jrheum.081088

[16] Wolff B Lodziewski S Bollmann T Opitz CF Ewert R. Impaired peripheral endothelial function in severe idiopathic pulmonary hypertension correlates with the pulmonary vascular response to inhaled iloprost. Am Heart J 2007 6;153(6):1088.e1–7. 17540215 10.1016/j.ahj.2007.03.005

[17] Zimmermann R Kreuder J Michel-Behnke I Voelkel NF Schranz D. Pulmonary flow reserve in children with idiopathic pulmonary arterial hypertension: implications for diagnosis and therapy. Eur J Med Res 2006;11:208–13. 16723295

[18] Ilsar R Chawantanpipat C Chan KH Dobbins TA Waugh R Hennessy A Measurement of pulmonary flow reserve and pulmonary index of microcirculatory resistance for detection of pulmonary microvascular obstruction. PLoS One 2010;5:e9601. 20231900 10.1371/journal.pone.0009601 PMC2834756

[19] van den Hoogen F Khanna D Fransen J Johnson SR Baron M Tyndall A 2013 classification criteria for systemic sclerosis: an American College of Rheumatology/European League against Rheumatism collaborative initiative. Arthritis Rheum 2013;65:2737–47. 24122180 10.1002/art.38098 PMC3930146

[20] Smith V Vanhaecke A Herrick AL Distler O Guerra MG Denton CP Fast track algorithm: How to differentiate a “scleroderma pattern” from a “non-scleroderma pattern”. Autoimmun Rev 2019 11;18(11):102394. 31520797 10.1016/j.autrev.2019.102394

[21] Soulaidopoulos S Triantafyllidou E Garyfallos A Kitas GD Dimitroulas T. The role of nailfold capillaroscopy in the assessment of internal organ involvement in systemic sclerosis: A critical review. Autoimmun Rev 2017;16:787–95. 28576600 10.1016/j.autrev.2017.05.019

[22] Arvanitaki A Giannakoulas G Triantafyllidou E Karvounis H Garyfallos A Kitas G Nailfold videocapillaroscopy: a novel possible surrogate marker for the evaluation of peripheral micro-angiopathy in pulmonary arterial hypertension. Scand J Rheumatol 2020 9 10;1–10. 10.1080/03009742.2020.1786854 32909481

[23] Riccieri V Vasile M Iannace N Stefanantoni K Sciarra I Vizza CD Systemic sclerosis patients with and without pulmonary arterial hypertension: a nailfold capillaroscopy study. Rheumatology (Oxford) 2013;52:1525–8. 23671125 10.1093/rheumatology/ket168

[24] Ohtsuka T Hasegawa A Nakano A Yamakage A Yamaguchi M Miyachi Y. Nailfold capillary abnormality and pulmonary hypertension in systemic sclerosis. Int J Dermatol 1997;36:116–22. 9109008 10.1046/j.1365-4362.1997.00088.x

[25] Smith V Riccieri V Pizzorni C Decuman S Deschepper E Bonroy C Nailfold capillaroscopy for prediction of novel future severe organ involvement in systemic sclerosis. J Rheumatol 2013;40:2023–8. 24128778 10.3899/jrheum.130528

[26] Xia Z Wang G Xiao H Guo S Liu Y Meng F Diagnostic value of nailfold videocapillaroscopy in systemic sclerosis secondary pulmonary arterial hypertension: a meta-analysis. Internal Medicine Journal 2018;48:1355–9. 29761614 10.1111/imj.13968

[27] Corrado A Correale M Mansueto N Monaco I Carriero A Mele A Nailfold capillaroscopic changes in patients with idiopathic pulmonary arterial hypertension and systemic sclerosis-related pulmonary arterial hypertension. Microvasc Res 2017;114:46–51. 28619664 10.1016/j.mvr.2017.06.005

[28] Hofstee HM Vonk Noordegraaf A Voskuyl AE Dijkmans BA Postmus PE Smulders YM Nailfold capillary density is associated with the presence and severity of pulmonary arterial hypertension in systemic sclerosis. Ann Rheum Dis 2009;68:191–5. 18375538 10.1136/ard.2007.087353

[29] Arvanitaki A Giannakoulas G Triantafyllidou E Feloukidis C Garyfallos A Karvounis H Assessment of peripheral microvascular changes in Eisenmenger syndrome: A nailfold video capillaroscopy study. In: 12th Advanced Symposium on Adult Congenital Heart Disease. Virtual Conference. 21–22 September 2020, London, UK.

[30] Arvanitaki A Giannakoulas G Triantafyllidou E Feloukidis C Boutou AK Garyfallos A Peripheral microangiopathy in pre-capillary pulmonary hypertension: a nailfold video capillaroscopy prospective study. Respiratory Research 2021;22. 33478514 10.1186/s12931-021-01622-1 PMC7819216

[31] Arvanitaki A Giannakoulas G Triantafyllidou E Karvounis H Dimitroulas T. Peripheral Microangiopathy in Patients with Precapillary Pulmonary Hypertension: Correlation with Cardiac Function and Patients’ Functional Capacity. Study Design and Rationale. Mediterr J Rheumatol 2020;31:369–73. 33163874 10.31138/mjr.31.3.369 PMC7641020

[32] Kielstein JT Bode-Boger SM Hesse G Martens-Lobenhoffer J Takacs A Fliser D Asymmetrical dimethylarginine in idiopathic pulmonary arterial hypertension. Arterioscler Thromb Vasc Biol 2005;25:1414–8. 15860741 10.1161/01.ATV.0000168414.06853.f0

[33] Boushel R Langberg H Olesen J Gonzales-Alonzo J Bulow J Kjaer M. Monitoring tissue oxygen availability with near infrared spectroscopy (NIRS) in health and disease. Scand J Med Sci Sports 2001;11:213–22. 11476426 10.1034/j.1600-0838.2001.110404.x

[34] Mancini DM Bolinger L Li H Kendrick K Chance B Wilson JR. Validation of near-infrared spectroscopy in humans. J Appl Physiol (1985) 1994;77:2740–7. 7896615 10.1152/jappl.1994.77.6.2740

[35] Panagiotou M Vogiatzis I Louvaris Z Jayasekera G MacKenzie A McGlinchey N Near infrared spectroscopy for the assessment of peripheral tissue oxygenation in pulmonary arterial hypertension. Eur Respir J 2016;48:1224–7. 27587562 10.1183/13993003.01022-2016

[36] Dimopoulos S Tzanis G Karabinis A Nanas S. Dynamic near-infrared spectroscopy assessment as an important tool to explore pulmonary arterial hypertension pathophysiology. Eur Respir J 2017 1 4;49(1):1602161. 28052959 10.1183/13993003.01932-2016

[37] Dimopoulos S Tzanis G Manetos C Tasoulis A Mpouchla A Tseliou E Peripheral muscle microcirculatory alterations in patients with pulmonary arterial hypertension: a pilot study. Respir Care 2013;58:2134–41. 23716709 10.4187/respcare.02113

[38] Fink C Puderbach M Bock M Lodemann KP Zuna I Schmahl A Regional lung perfusion: assessment with partially parallel three-dimensional MR imaging. Radiology 2004;231:175–84. 15068947 10.1148/radiol.2311030193

[39] Hatabu H Gaa J Kim D Li W Prasad PV Edelman RR. Pulmonary perfusion: qualitative assessment with dynamic contrast-enhanced MRI using ultra-short TE and inversion recovery turbo FLASH. Magn Reson Med 1996;36:503–8. 8892200 10.1002/mrm.1910360402

[40] Ley S Kauczor HU. MR imaging/magnetic resonance angiography of the pulmonary arteries and pulmonary thromboembolic disease. Magn Reson Imaging Clin N Am 2008;16:263–73. 18474331 10.1016/j.mric.2008.02.012

[41] Kreitner KF Kunz RP Ley S Oberholzer K Neeb D Gast KK Chronic thromboembolic pulmonary hypertension - assessment by magnetic resonance imaging. Eur Radiol 2007;17:11–21. 16838142 10.1007/s00330-006-0327-x

[42] Kauczor HU Ley-Zaporozhan J Ley S. Imaging of pulmonary pathologies: focus on magnetic resonance imaging. Proc Am Thorac Soc 2009;6:458–63. 19687219 10.1513/pats.200901-002AW

[43] Ohno Y Hatabu H Murase K Higashino T Kawamitsu H Watanabe H Quantitative assessment of regional pulmonary perfusion in the entire lung using three-dimensional ultrafast dynamic contrast-enhanced magnetic resonance imaging: Preliminary experience in 40 subjects. J Magn Reson Imaging 2004;20:353–65. 15332240 10.1002/jmri.20137

[44] Anwar A Ruffenach G Mahajan A Eghbali M Umar S. Novel biomarkers for pulmonary arterial hypertension. Respir Res 2016;17:88. 27439993 10.1186/s12931-016-0396-6 PMC4955255

[45] Dimitroulas T Giannakoulas G Papadopoulou K Sfetsios T Karvounis H Dimitroula H Left atrial volume and N-terminal pro-B type natriuretic peptide are associated with elevated pulmonary artery pressure in patients with systemic sclerosis. Clin Rheumatol 2010;29:957–64. 20526641 10.1007/s10067-010-1494-3

[46] Dimitroulas T Giannakoulas G Sfetsios T Karvounis H Dimitroula H Koliakos G Asymmetrical dimethylarginine in systemic sclerosis-related pulmonary arterial hypertension. Rheumatology (Oxford) 2008;47:1682–5. 18753191 10.1093/rheumatology/ken346

[47] Giannakoulas G Mouratoglou SA Gatzoulis MA Karvounis H. Blood biomarkers and their potential role in pulmonary arterial hypertension associated with congenital heart disease. a systematic review. Int J Cardiol 2014;174:618–23. 24814894 10.1016/j.ijcard.2014.04.156

[48] Ho JE Liu C Lyass A Courchesne P Pencina MJ Vasan RS Galectin-3, a marker of cardiac fibrosis, predicts incident heart failure in the community. J Am Coll Cardiol 2012;60:1249–56. 22939561 10.1016/j.jacc.2012.04.053 PMC3512095

[49] Fenster BE Lasalvia L Schroeder JD Smyser J Silveira LJ Buckner JK Galectin-3 levels are associated with right ventricular functional and morphologic changes in pulmonary arterial hypertension. Heart Vessels 2016;31:939–46. 25976729 10.1007/s00380-015-0691-z

[50] Li LC Li J Gao J. Functions of galectin-3 and its role in fibrotic diseases. J Pharmacol Exp Ther 2014;351:336–43. 25194021 10.1124/jpet.114.218370

[51] Malhotra R Paskin-Flerlage S Zamanian RT Zimmerman P Schmidt JW Deng DY Circulating angiogenic modulatory factors predict survival and functional class in pulmonary arterial hypertension. Pulm Circ 2013;3:369–80. 24015338 10.4103/2045-8932.110445 PMC3757832

[52] Tiede SL Gall H Dorr O dos Santos Guilherme M Troidl C Liebetrau C New potential diagnostic biomarkers for pulmonary hypertension. Eur Respir J 2015;46:1390–6. 26250494 10.1183/13993003.00187-2015

[53] Diller GP van Eijl S Okonko DO Howard LS Ali O Thum T Circulating endothelial progenitor cells in patients with Eisenmenger syndrome and idiopathic pulmonary arterial hyper-tension. Circulation 2008;117:3020–30. 18519847 10.1161/CIRCULATIONAHA.108.769646

[54] Levy M Bonnet D Mauge L Celermajer DS Gaussem P Smadja DM. Circulating endothelial cells in refractory pulmonary hypertension in children: markers of treatment efficacy and clinical worsening. PLoS One 2013;8:e65114. 23762293 10.1371/journal.pone.0065114 PMC3677895

[55] Smadja DM Gaussem P Mauge L Israel-Biet D Dignat-George F Peyrard S Circulating endothelial cells: a new candidate biomarker of irreversible pulmonary hypertension secondary to congenital heart disease. Circulation 2009;119:374–81. 19139384 10.1161/CIRCULATIONAHA.108.808246

[56] Damico R Kolb TM Valera L Wang L Housten T Tedford RJ Serum endostatin is a genetically determined predictor of survival in pulmonary arterial hypertension. Am J Respir Crit Care Med 2015;191:208–18. 25489667 10.1164/rccm.201409-1742OC PMC4347439

[57] Kumpers P Nickel N Lukasz A Golpon H Westerkamp V Olsson KM Circulating angiopoietins in idiopathic pulmonary arterial hypertension. Eur Heart J 2010;31:2291–300. 20601390 10.1093/eurheartj/ehq226

[58] Yang D Liu Z Yang Z. Ghrelin and its relation with N-terminal brain natriuretic peptide, endothelin-1 and nitric oxide in patients with idiopathic pulmonary hypertension. Cardiology 2013;124:241–5. 23571554 10.1159/000348368

[59] Santos-Ferreira CA Abreu MT Marques CI Goncalves LM Baptista R Girao HM. Micro-RNA Analysis in Pulmonary Arterial Hypertension: Current Knowledge and Challenges. JACC Basic Transl Sci 2020;5:1149–62. 33294743 10.1016/j.jacbts.2020.07.008 PMC7691282

[60] Sarrion I Milian L Juan G Ramon M Furest I Carda C Role of circulating miRNAs as biomarkers in idiopathic pulmonary arterial hypertension: possible relevance of miR-23a. Oxid Med Cell Longev 2015;2015:792846. 25815108 10.1155/2015/792846 PMC4357130

[61] Perros F Dorfmuller P Montani D Hammad H Waelput W Girerd B Pulmonary lymphoid neogenesis in idiopathic pulmonary arterial hypertension. Am J Respir Crit Care Med 2012;185:311–21. 22108206 10.1164/rccm.201105-0927OC

